# Identification of an Active New *Mutator* Transposable Element in Maize

**DOI:** 10.1534/g3.111.000398

**Published:** 2011-09-01

**Authors:** Bao-Cai Tan, Zongliang Chen, Yun Shen, Yafeng Zhang, Jinsheng Lai, Samuel S. M. Sun

**Affiliations:** * Institute of Plant Molecular Biology and Agricultural Biotechnology, School of Life Sciences, State Key Lab of Agrobiotechnology, The Chinese University of Hong Kong, Shatin, New Territory Hong Kong, and; † State Key Lab of Agrobiotechnology, China Agricultural University, Beijing, 100094 China

**Keywords:** *Mutator* transposon, *Mutator13*, mutagenesis, maize, transposon tagging, cloning

## Abstract

Robertson’s *Mutator* (*Mu*) system has been used in large scale mutagenesis in maize, exploiting its high mutation frequency, controllability, preferential insertion in genes, and independence of donor location. Eight Mutator elements have been fully characterized (*Mu1*, *Mu2* /*Mu1*.*7*, *Mu3*, *Mu4*, *Mu5*, *Mu6/7*, *Mu8*, *MuDR*), and three are defined by TIR (*Mu10*, *Mu11* and *Mu12*). The genome sequencing revealed a complex family of Mu-like-elements (MULEs) in the B73 genome. In this article, we report the identification of a new *Mu* element, named *Mu13*. *Mu13* showed typical *Mu* characteristics by having a ∼220 bp TIR, creating a 9 bp target site duplication upon insertion, yet the internal sequence is completely different from previously identified *Mu* elements. *Mu13* is not present in the B73 genome or a *Zea mays* subsp. *parviglumis* accession, but in W22 and several inbreds that found the Robertson’s *Mutator* line. Analysis of mutants isolated from the UniformMu mutagenic population indicated that the *Mu13* element is active in transposition. Two novel insertions were found in expressed genes. To test other unknown *Mu* elements, we selected six new *Mu* elements from the B73 genome. Southern analysis indicated that most of these elements were present in the UniformMu lines. From these results, we conclude that *Mu13* is a new and active *Mu* element that significantly contributed to the mutagenesis in the UniformMu population. The Robertson’s *Mutator* line may harbor other unknown active *Mu* elements.

*Mutator (Mu*) transposable elements are a major class of class II transposons identified in maize by Donald Robertson (1978, 1981). The two-component system, one autonomous *MuDR* and many nonautonomous *Mu* elements, was exploited for efficient mutagenesis in maize. High copies of the elements offer a high forward mutation frequency, whereas limited copies of *MuDR* allowed turning off the transposition by removing the element through segregation (McCarty *et al.* 2005). Preferential transposition into gene rich regions by *Mu* elements enhances mutagenesis frequency. And transposition not limited to linked loci facilitates genome wide mutagenesis. For these reasons, several mutant populations in maize were created by using the *Mu* system (Bensen *et al.* 1995; May *et al.* 2003; Raizada 2003; McCarty *et al.* 2005).

The well-characterized *Mu* elements (*Mu1* to *Mu9/MuDR*) were discovered exclusively in maize. Subsequent molecular analyses and genome sequencing revealed that *Mu* elements are present in plants (Lisch 2002), fungi (Chalvet *et al.* 2003), bacteria (Eisen *et al.* 1994), protozoans (Pritham *et al.* 2005), and metazoans (Hua-Van and Capy 2008). Based on sequence similarity, these elements are classified as Mu-like elements (MULEs). MULEs belong to a superfamily of transposons with complex members and diverse sequences. Typical characteristics of this family include a conserved 50–200bp terminal inverted repeat (TIR), unrelated internal sequences between the TIRs, and creating a 9bp target site duplication (TSD). In contrast, all the previously identified *Mu* elements from maize (*Mu1-Mu9/MuDR*) carry a ∼220bp TIR that is highly conserved. Transposition activity of the elements is thought to be associated with the TIR sequences. Inactive elements carry mutated TIRs.

Different from *Ac/Ds* and *Spm/dSpm* transposable elements where the non-autonomous elements are deletion derivatives of the autonomous elements, the internal sequences between TIRs among *Mu* elements are often unrelated. Some *Mu* internal sequences showed high similarity to host genome, suggesting a possible gene capture in the formation of these elements. This class of *Mu* elements was classified as Pack-MULEs (Jiang *et al.* 2004). About 262 Pack-MULEs were identified in the B73 genome (Schnable *et al.* 2009). Because promoters are found in the TIRs, *Mu* internal sequences may be transcribed in convergent orientations (Hershberger *et al.* 1995; Lisch 2002). Hence, it was suspected that some of the Pack-MULEs may have regulatory function, as antisense transcripts may interfere with expression of the endogenous genes (Lisch 2005; Juretic *et al.* 2005).

Transposition of all *Mu* elements required the presence of an active *MuDR* element. The *MuDR* element contains two genes, *mudrA* encoding a transposase (MURA) and *mudrB* whose product (MURB) is of unknown function. MURA showed high similarity to bacterial transposase and the virus integrase (Walbot and Rudenko 2002); hence, it is essential for transposition. Transposable elements containing only *mudrA*-like genes were found in both monocots and eudicots (Saccaro *et al.* 2007). The *mudrB* gene is only present in the genus *Zea* (Lisch *et al.* 2001). *Jittery*, an autonomous transposon identified in maize, contains a *mudrA*-like gene, but with TIR sequences distinct from *Mu* elements (Xu *et al.* 2004). *Jittery* exhibited high frequency of excision, causing somatic and germinal reversion, but apparently lost its activity for new insertions. Transposition of *Mu* elements employs two distinct mechanisms. In somatic cells, transposition mostly uses a “cut-and-paste” mechanism. The element cuts itself and reinserts it in a new locus elsewhere in the genome. High-frequency excision of *Mu* elements is restricted to the late stage of cells in development during organogenesis. In germinal cells, *Mu* transposition uses a “replicate-and-insert” mechanism where the element replicates just before meiosis or in the gametophytes and inserts in a new locus in the genome. Consequently, “cut and paste” transposition does not increase the copy number, whereas “duplicate-and-insert” transposition does. Excision of a *Mu* element left a footprint of the 9bp TSD, which sometimes restored the function of the donor gene such as in *bz1-mum9* (McCarty *et al.* 2005).

Prior to the sequencing of the maize genome, eleven *Mu* elements were reported in maize, of which eight were characterized by full sequences, (*i.e.*, *Mu1*, *Mu2* /*Mu1*.*7*, *Mu3*, *Mu4*, *Mu5*, *Mu6/7*, *Mu8* and *MuDR*) (Bennetzen *et al.* 1984; Taylor and Walbot 1987; Talbert *et al.* 1989; Fleenor *et al.* 1990), and three were indicated by TIRs (Dietrich *et al.* 2002). The sequencing of the B73 genome revealed a surprisingly complex view of the *Mutator* family, which accounts for approximately 1% of the 2.3 gbp genome (Schnable *et al.* 2009). These include MULEs, Pack-MULEs, and SOLOs that contain only one TIR. Many of these elements contain a shorter TIR, suggesting that these elements may have lost the capacity for transposition. In this study, we report a new *Mu* element, *Mu13*, which was identified from the UniformMu population, a derivative *Mu* active line from the Robertson’s *Mu* line. *Mu13* exhibits typical *Mu* characteristics and is active in transposition. It contributes significantly to mutagenesis. The finding of *Mu13* adds to the active *Mu* reservoir and facilitates cloning of causative insertions in the *Mu* tagged mutants in phenotype-driven forward genetics in maize.

## Materials and Methods

### Genetic stocks

The maize lines used in this study were derived from the UniformMu population, a *Mutator* line with the mutable *bz1-mum9* anthocyanin biosynthetic gene introgressed into the W22 genetic background (McCarty *et al.* 2005). The teosinte lines *Zea mays* subsp*. parviglumis* (Accession: PI 384061) and *Zea mays* subsp. *mexicana* (Accession: PI 566684) were provided by the Maize Genetic Stock Center. Other inbred lines (W22, B73, Mo17, M14, Q66, Q67, B77, and B79) were generously provided by Donald R. McCarty (University of Florida).

### Cloning of Mu13 from UniformMu population

The *Mu13* transposable element was amplified by a pair of primers (5′-CTGCTCCTGTGCTATCCTCC-3′ and 5′-ACCAAACCAACAAGAGCCTG-3′) flanking a *Mu13* insertion in a gene coding for a putative plastid Sigma factor3 (*ZmSig3*). Template DNA was isolated from line 03S-4081-01, homozygous for the insertion. As *Mu* elements carry a long terminal inverted repeat (∼220bp), it interferes with PCR amplification. We tested different conditions with DNA polymerases of various sources. ExTaq (TaKaRa, Japan) and ThermalAce DNA polymerases (Invitrogen, USA) yielded successful amplification. The PCR reaction was composed of 20mm Tris-HCl pH 8.4, 50 mm KCl, 2mm MgCl_2_, 200 μm of each dNTP, 100 nM each primer, 5% DMSO, and 1 U of DNA polymerase. PCR conditions were 96°C/3min for initial denaturation, 8 cycles (95°C/30 sec, 62°C/30 sec, 72°C/2min) followed by 30 cycles (95°C/30 sec, 58°C/30sec, 72°C/2min), with final extension at 72°/10min. The PCR fragment was purified from gel by gel extraction kit (Zymo Research, USA), ligated into pCR4-TOPO (Invitrogen, USA), and sequenced.

#### Selection and cloning of new Mu elements in B73:

A conserved 200bp *Mu* TIR sequence based on known *Mu* elements (*Mu1* to *Mu9/ MuDR*) was used in a BLAST search of the GenBank maize sequences, with a cut-off E value of < e^−10^. Within this collection, the known *Mu* elements were identified by a BLAST search with the internal sequence of each *Mu* element. Identical sequences were clustered using BLASTCLUST (ftp://ftp.ncbi.nih.gov/blast). The resulting collection was analyzed for left- and right-TIR in terms of orientation and homology, as well as the presence/absence of a 9bp host sequence direct duplication.

We amplified the internal sequences of six new *Mu* elements that carry highly conserved TIR at both ends. The primers were listed in Table I, and the PCR conditions were similar to those present in the amplification of *Mu13*. The internal sequences were cloned in pCR4-TOPO and sequenced.

#### Selection of UniformMu mutants for Southern blot analyses:

UniformMu mutants segregating for visible mutant phenotype of embryo defective (emb), small kernel (smk), empty pericarp (emp), shrunken (sh), and defective kernel (dek) were randomly chosen. The 18 mutants were 06S-6001 (smk), 06S-6002 (emp); 06S-6004 (defective kernel, dek); 06S-6005 (emp); 06S-6016 (smk); 06S-6018 (dek); 06S-6019 (smk); 06S-6020 (emb); 06S-6023 (emp); 06S-6026 (smk/dek); 06S-6029 (smk); 06S-6032 (smk); 06S-6033 (emb); 06S-6034 (dek); 06S-6044 (dek); 06S-6045 (emp); and 06S-6055 (sh/smk). Each DNA was extracted from seedlings of three individual ears that were genotyped based on the seed phenotype. All these ears did not exhibit active *MuDR* activity, as indicated by the mutable *bz1-mum9* anthocyanin biosynthetic marker. All these lines were back-crossed with W22 twice.

#### DNA extraction and Southern analysis:

Genomic DNA was isolated by a urea–phenol–chloroform-based method. 1g fresh weight of leaf tissues was ground in liquid nitrogen and extracted with 5 ml of DNA extraction buffer (7 M urea, 0.3 M NaCl, 50 mM Tris-HCl, 24 mM EDTA, and 1% sarkosine, pH 8.0). After mixing with 4 ml phenol–chloroform–isoamyl alcohol (25:24:1), the extraction was carried out with gentle shaking for 30 min at room temperature. The mixture was separated by centrifugation at 4800 × g for 15 min. The aqueous phase was transferred to a new tube and mixed with 0.1 volume of 3M sodium acetate (pH 5.2) and 3.8 ml isopropanol. DNA was pelleted at 4800 × g for 5 min, washed with 70% ethanol, and dissolved in TE buffer (10 mM Tris-HCl, 1 mM EDTA, pH 8). Approximately 10 µg genomic DNA was digested with appropriate restriction enzymes at 37°C for 6 hr. The DNA was resolved on a 0.7% agarose gel, denatured, and blotted onto a Hybond-N membrane (GE Healthcare). The membrane was cross-linked and hybridized. The probe was labeled with Ready-To-Go DNA labeling beads and purified with ProbeQuant G-50 micro column (GE Healthcare).

The probes used for Southern analyses for *Mu14*–*Mu19* were amplified from the B73 genome by PCR with primers listed in Table 1. The primer anchor positions with respect to TIR and probe sequences are listed in supporting information, File S1. For *Mu13*, it was derived from UniformMu by PCR with a single primer (5′-ATCAATGTCCTGTCACCGTTTACCGT-3′) that was anchored in the TIR region.

**Table 1:** Primers used in amplification of selected *Mu* elements in B73

Primer	Sequence
Mu14-iF1	5′-CTCTTCCCCACACCTATTGC-3′
Mu14-iR1	5′-GAGATGCTCCGCGATTACAT-3′
Mu15-iF1	5′-TAAGGTGATTTGCTCGGGTC-3′
Mu15-iR1	5′-TCTCTTGCTTCTCCGTCTCC-3′
Mu16-iF1	5′-CACCGTCAGGCTTAACAACA-3′
Mu16-iR1	5′-CGGTGAGTTCTCCTCCTCTG-3′
Mu17-iF1	5′-CTCAGCGAACTCTGGCACAC-3′
Mu17-iR1	5′-CACTCCTCTCCGTCTCCGAT-3′
Mu18-iF1	5′-TTGGAGGTGTCGGTAGTGAGC-3′
Mu18-iR1	5′-ACAGCTCTTGCGTCTCCTCTG-3′
Mu19-iF1	5′-ATTGGAGTGCTCTCGGGGT-3′
Mu19-iR1	5′-AGAGCTCGGTCTCAGGCATTA-3′

#### Bioinformatics analysis:

Sequence alignments were carried out using the CLUSTALW algorithm available online (http://workbench.sdsc.edu/). For phylogenetic tree construction, the phylogenetic tree files from CLUSTALW analysis were imported into a TreeView program (http://taxonomy.zoology.gla.ac.uk/rod/treeview.html).

## Results

### Identification of *Mu13* element

**Figure 1:**
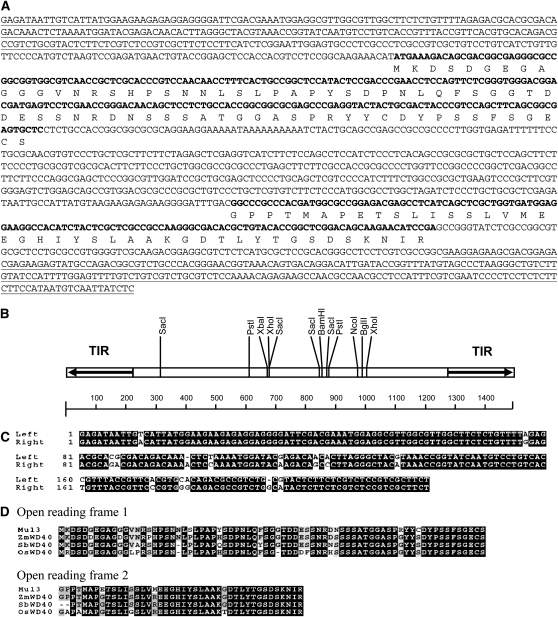
Sequence characteristics of *Mu13* element. (A) Sequences of *Mu13*. Terminal-inverted repeat (TIR) region is underlined. Bold sequences indicated two open reading frames and conceptual translation. (B) Structure and restriction map of *Mu13*. (C) Alignment of *Mu13* left and right TIR. (D) Alignment of two ORFs of *Mu13* element with three WD40 proteins, maize ZmWD40 (ACG25371), sorghum SbWD40 (Sb01g008680), and rice OsWD40 (Os03g0738700).

In a large scale extraction of *Mu* flanking sequences from mutants isolated from the UniformMu population (McCarty *et al.* 2005), a *Mu* element was found inserted in a gene coding for a putative plastid Sigma factor 3 (*ZmSig3*). The element was inserted in the third exon of the gene (refer to Figure 4, Accession no. CG893004). We cloned the *Mu* element and found that the element is 1494bp long, containing a 223bp TIR with an 88% identity to the consensus of previously known *Mu* TIRs (Figure 1). The left and right TIR showed a higher identity (92%), which is a general feature of the *Mu* elements. A 9bp direct target site duplication was found at the insertion site. The internal sequence of this element is completely different from any previously identified *Mu* elements (Bennetzen 1984; Taylor & Walbot 1987; Chen *et al.* 1987; Talbert *et al.* 1989; Fleenor *et al.* 1990; Hershberger *et al.* 1991). Searching the GenBank and the nearly completed B73 genome did not find the presence of this element. In light of the partially characterized *Mu* elements, *Mu10*, *Mu11*, *and Mu12* (Dietrich *et al.* 2002), we designated this element as *Mu13* (Accession: HQ698272).

Bioinformatic analysis revealed that the *Mu13* internal sequences contain two open reading frames (ORF). The conceptually translated protein sequences of these two ORFs showed high similarity to a maize protein that was annotated as nucleotide binding protein (accession: ACG25371, GRMZM2G317614). Further analysis revealed that it encodes a WD40 protein, containing seven WD repeats. As indicated in Figure 1D, the first highly similar ORF started from the first methionine and covered 73 amino acid (aa) residues in length. This region shared an 88% identity with the maize WD40 protein, and a similar identity with apparent orthologs in sorghum (Sb01g008680) and rice (Os03g0738700, also identified as Os03g52870, annotated as transducin family protein). The second highly similar region (95% identity) was about 42 aa long and coincided with the first repeat of the WD40 protein. In the maize WD40 protein, these two regions were separated by 26 amino acid residues, which were not found in the *Mu13*. This maize WD40 gene was expressed as indicated by ESTs, suggesting that it may be a functional gene. Another WD40 gene on maize chromosome 5 (GRMZM5G852097) is apparently a syntentic paralogous duplicate of GRMZM2G317614, which is also probably functional.

### *Mu13* is active in the UniformMu population

**Figure 2:**
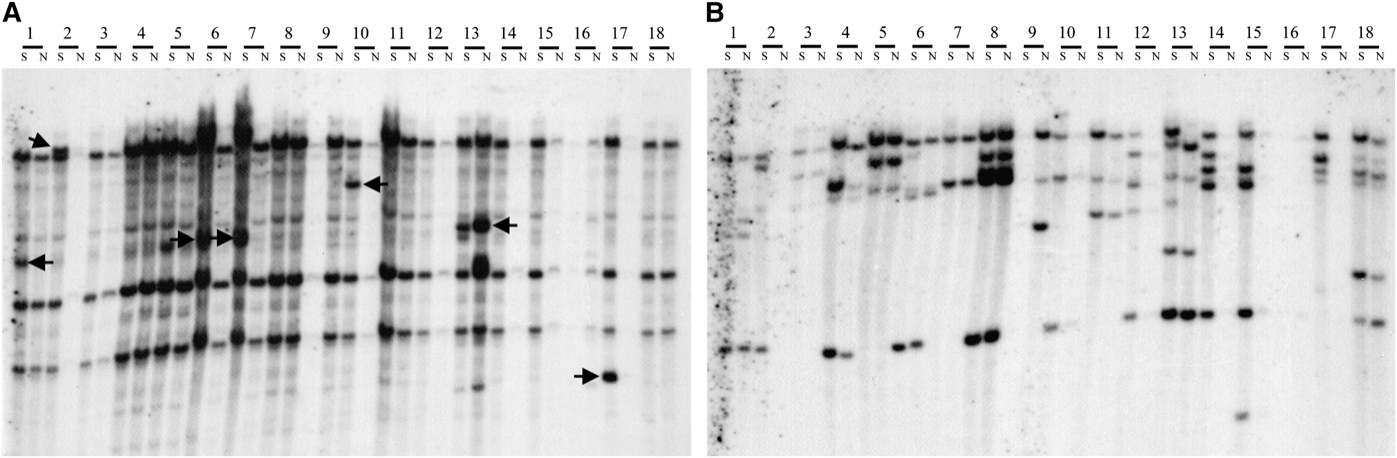
Detection of new transpositions of the *Mu13* element in the UniformMu population. Genomic DNAs from eighteen randomly selected lines that segregate different seed mutant phenotypes were digested with *Eco*RI and hybridized with a *Mu13* (A) and a *Mu1* specific probe (B). A pooled WT (non-segregant, N) and a segregant (segregating each mutant phenotype, S) sample were used from each line (refer to *Materials and Methods*). Arrows indicate *Mu13* insertions that were not found in the progenitors.

The insertion of *Mu13* in a functional gene in the UniformMu population suggested that it was active in transposition. This insertion was not present in the parental lines that gave rise to the mutant. It is known that *Mu* elements are not equally active. *Mu4*, *Mu5*, and *Mu7* were less active than the other known ones (Talbert *et al.* 1989), and so far most genes cloned by transposon tagging were inserted by *Mu1*/*2*, *Mu3*, *Mu8*, and *MuDR*. To assess the *Mu13* transposition activity, we analyzed 18 UniformMu seed phenotype mutants randomly selected from a large set of available seed phenotype mutants. For each mutant, seeds showing no *MuDr* activity (lack of somatic transposition indicated by the *bz1-mum9* marker gene) were selfed to produce an F2 mutant segregating family. The genotype of each F2 individual was scored by examining the ear. DNAs from three F2 individuals of either wild type (not segregating mutant phenotypes, N) or segregating seed mutant phenotype (S) were pooled separately and analyzed by Southern hybridization. As shown in Figure 2, hybridization with a *Mu13* probe detected seven new *Mu13* insertions, as indicated by the appearance of new *Mu13* containing fragments. Because the *Mu13* probe used in this analysis contained 80bp TIR sequences, it cross-hybridized with related *Mu* elements and produced weak signals. The *Mu13* signals were strong. Three *Mu13* containing fragments (4.5kb, 5.8kb, >12kb) showed uniform presence in all the members, suggesting that they are apparently parental. When the same blot was hybridized with a *Mu1*/*Mu2* specific probe, comparable numbers of *Mu1/Mu2* insertions were detected (Figure 2B). Some of these insertions were unique to individual lines, suggesting new transposition by *Mu1/Mu2* as well. This result indicated that *Mu13* is active in the UniformMu population.

### *Mu13* presence in maize inbred lines and teosinte

**Figure 3:**
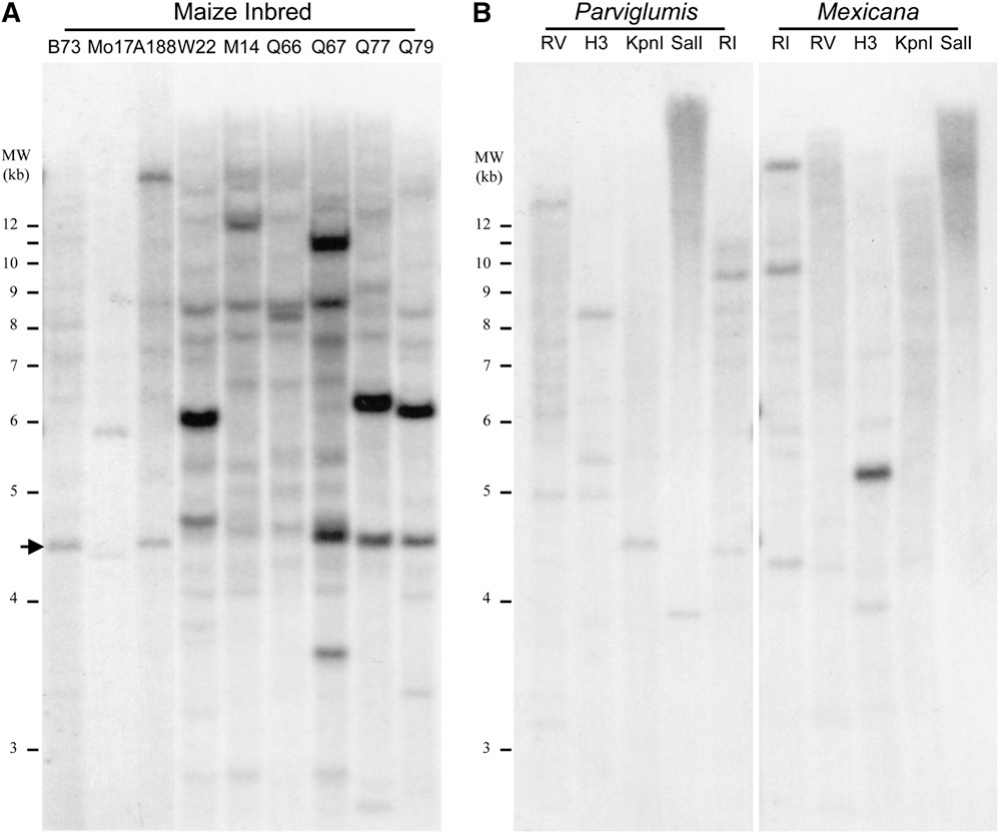
Southern analysis of *Mu13* element presence in teosinte and maize lines. (A) Genomic DNAs of selected maize inbreds were digested with *EcoRI* and hybridized with a *Mu13* probe. The arrow indicates the 4382bp *EcoRI* fragment of the WD40 gene (Accession no. ACG25371). (B) Genomic DNAs from *Zea mays* subsp*. parviglumis* (Accession no. PI 384061) and *Zea mays* subsp. *mexicana* (Accession no. PI 566684) were digested with five different restriction enzymes (RV: *EcoRV*, H3: *HindIII*, *KpnI*, *SalI*, RI: *EcoRI*) and hybridized with a *Mu13* probe.

The UniformMu mutagenic population was derived from introgressing Robertson’s *Mu*-active line into W22 genetic background (McCarty *et al.* 2005). Hence, the *Mu13* element can be derived from either W22 or Robertson’s *Mu*-active line. To determine the presence of *Mu13*, nine inbred lines of maize were analyzed by Southern blot analysis by using the *Mu13* internal sequence as a probe. Six inbred lines (W22, M14, Q66, Q67, Q77, and Q79) that founded the Robertson’s *Mutator* population were included. To ensure a complete digestion, *EcoRI* was used, because it is methylation insensitive and does not cut *Mu13* internally (Figure 1B). As shown in Figure 3A, *Mu13* was detected in W22, Q67, Q77, and Q79, and was not detected in B73, Mo17, or A188. *Mu13* was probably not present in M14 and Q66 because the hybridized bands were substantially weak in comparison to other lines. PCR analysis by using *Mu13* specific primers did not detect *Mu13* in B73, Mo17, A188, M14, and Q66, suggesting that the weak signal may have arisen from non-specific hybridization with the probe. W22 appeared to contain two copies of *Mu13*, whereas other inbred lines contained one to two copies. In B73, a ∼4.4kb fragment was weakly hybridized. This fragment is consistent with a WD40 gene (Accession no. ACG25371), which predicts a 4382 kb *EcoRI* fragment. The fragment contained a 258bp region that has 95% identity, and a 126bp region that has 98% identity to the *Mu13* probe. It was expected to weakly hybridize with the *Mu13* probe. In Mo17, the corresponding fragment is predicted to be 4302bp which was cross-hybridized as well. The *Mu13* probe carried an 80bp sequence of the TIR (refer to File S1), which predictably would weakly hybridize with related *Mu* elements. This might explain the background and weak signals.

To test whether *Mu13* is present in the ancestor of maize, we analyzed *Zea mays* subsp. *parviglumis* (Accession no. PI 384061) and *Zea mays* subsp. *mexicana* (Accession no. PI 566684). The former is believed to be the ancestor of maize from a single domestication process (Matsuoka *et al.* 2002). To reduce the chance that *Mu13* may have resulted from a large fragment that escaped from Southern detection, five restriction enzymes (*EcoRI*, *EcoRV*, *HindIII*, *KpnI* and *SalI*) that did not digest inside *Mu13* were used to restrict the genomic DNA. *Zea mays* subsp. *parviglumis* did not contain any *Mu13* element, as indicated by the absence of a hybridized signal (Figure 3B). A 5kb *HindIII* fragment was detected in *Zea mays* subsp. *mexicana*, but the signal intensity was much weaker than the *Mu13* signals in W22 or Q79. Because this hybridization was carried out under the same conditions at which the inbred DNAs were hybridized (and the loading was comparable with samples such as W22 or Q79), the signal produced in *Zea mays* subsp*. mexicana* was more likely from the WD40 fragment or an unknown homologous fragment than the real *Mu13* element. Although the primers were proven robust, subsequent PCR detection by *Mu13*-specific primers failed to amplify the *Mu13* element from *Zea mays* subsp*. mexicana*, indicating that *Mu13* was not present in *Zea mays* subsp. *mexicana* either. This result indicated that *Mu13* is not present in the sample of two teosinte accessions tested, but as substantial genetic diversity exists among teosinte accessions, we cannot infer its absence among all teosintes.

### Insertions of *Mu13* in functional genes

**Figure 4:**
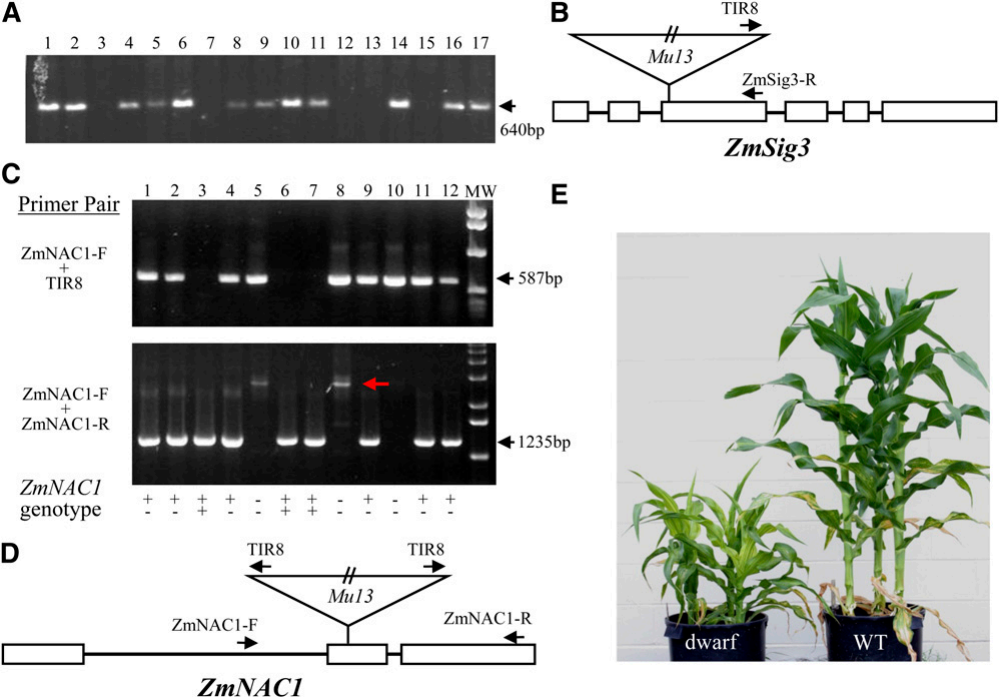
Insertion of *Mu13* element into a gene coding for putative plastid sigma factor 3 (*ZmSig3*) and an NAC domain-containing protein (ZmNAC1). (A) PCR segregation analysis of *ZmSig3* mutant segregation population carrying a *Mu13* element insertion. 1–17 were individual plants of an F2 family. The primers were TIR8-specific for *Mu* ends and ZmSig3-R–specific for *ZmSig3*. (B) Structure of the *ZmSig3* gene. Exons were designated as boxes and introns as lines. The *Mu13* insertion and primer anchor sites are indicated. The *Mu13* element is not drawn to scale. (C) PCR genotyping of a segregation population of the *Mu13* insertion in *ZmNAC1*. 1–12 were individual plants of the population. ZmNAC1-F and ZmNAC1-R are primers specific to *ZmNAC1*. ++, +−, and − − designated WT, heterozygote and homozygote for the insertion in *ZmNAC1* gene, respectively. The red arrow indicates a fragment containing a *Mu13* element. (D) Structure of the *ZmNAC1* gene and primer locations. (E) Plants of #5, #8, and #10 (in C) exhibited a dwarf plant phenotype.

A *Mu13* insertion was first identified in molecular characterization of the *ZmSig3* gene (Accession no. CG893004, GRMZM5G830932). The *ZmSig3* gene consists of six exons, and the *Mu13* was inserted in the third exon (Figure 4B). Analysis of the progenitor lines and a population segregating *zmsig3* mutants by PCR using Mu-TIR primer (TIR8) and the *ZmSig3* specific primer (ZmSig3-R) indicated that this insertion was not present in WT and the progenitor lines, suggesting that it was a new transposition event.

The insertion in *ZmSig3* suggested that the *Mu13* element may contribute significantly to mutagenesis in the UniformMu population. In a previous study on seed mutants isolated from the UniformMu mutagenesis, Mu-flanking sequences were extracted by the Mu-TAIL method (deposited in GenBank, McCarty *et al.* 2005). The identity of the *Mu* element, however, is unknown. To search for insertions by *Mu13*, we chose two *Mu* insertions in known functional genes for analysis. Analysis of the *Mu* flanking sequences indicated that one *Mu* element was inserted in a paralog of *Vp14* gene on chromosome 5S, named as *Vp14b* (GRMZM5G838285), and that the other was inserted in a gene coding for an NAC (NAM, ATAF1,2, CUC2) transcription factor domain containing protein, named ZmNAC1 (GRMZM2G312201). Both insertions were novel, as they were not present in the progenitors and were segregated specifically in individual lines. We cloned and sequenced the inserted *Mu* elements. The insertion in *Vp14b* was a *Mu7* element (data not shown), and the one in ZmNAC1 was a *Mu13* element (Figure 4D). We analyzed a population of 12 individual plants derived from a selfed heterozygote of the *Mu13* insertion by using *Mu* TIR specific primer TIR8 and *ZmNAC1* specific primers. The 12 individual plants were genotyped (Figure 4C). Plants homozygous for the *Mu13* insertion showed a dwarf phenotype (#5, #8, and #10 in Figure 4E). Although the insertion was not confirmed as the cause of the dwarf phenotype, it indicated at least a linkage between this *Mu13* insertion and the dwarf phenotype. The *ZmNAC1* is likely a functional gene, as multiple ESTs were found in GenBank.

### Presence and transposition of six new *Mu* elements in UniformMu lines

The sequencing of the maize genome revealed a surprising view of the *Mutator* family, which accounts for 1% of the B73 genome (Schnable *et al.* 2009). We used the conserved 200bp *Mu* TIR sequences and performed a BLAST search of the maize genomic sequences in GenBank. A high stringency search (E value < e^−10^) resulted in a low return of *Mu* elements. It appears that four types of *Mu* elements with distinct TIRs are present in the B73 genome. One class of *Mu* elements possesses TIRs with high similarity to known *Mu* elements in sequence and length (left and right TIR ∼210 bp). A second class contains a left TIR of ∼215bp and a short right TIR of ∼90bp. A third class contains both short TIRs (∼100bp), and a fourth class is called SOLOs, which contain only one TIR. The previously known *Mu* elements only account for a very small fraction of this family. *Mu1*, *Mu2*, *Mu8*, and *Mu13* do not exist in the B73 genome. B73, however, does contain truncated and apparently non-functional derivatives of the autonomous *MuDR*, as well as one copy of *Mu3* and *Mu7*, two copies of *Mu4* and *Mu5*, and four copies of *Mu7* derivatives that have insertions or deletions in their internal sequences. Because of the absence of *MuDR*, these elements are dormant, and some may have lost their transposition activity due to accumulated mutations. To analyze the presence and possible activity of the unknown *Mu* elements in the UniformMu population, we identified a subset of MULEs from the B73 genome. The criteria are that the element *1*) contains a highly conserved ∼220bp TIR on both ends (>85% identity to consensus *Mu* TIR sequence); *2*) contains perfect TIR ends (GAGATA at the 5′ and TATCTC at 3′); and *3*) possesses perfect TSD in the insertion site, which is indicative for recent transposition. The known active *Mu* elements all contain these features. We chose six MULEs that showed the highest similarity to *Mu* TIR consensus and with unrelated internal sequences. These elements were named as *Mu14* to *Mu19* (Accessions no. HQ698273–HQ698278, refer to File S1).

**Figure 5:**
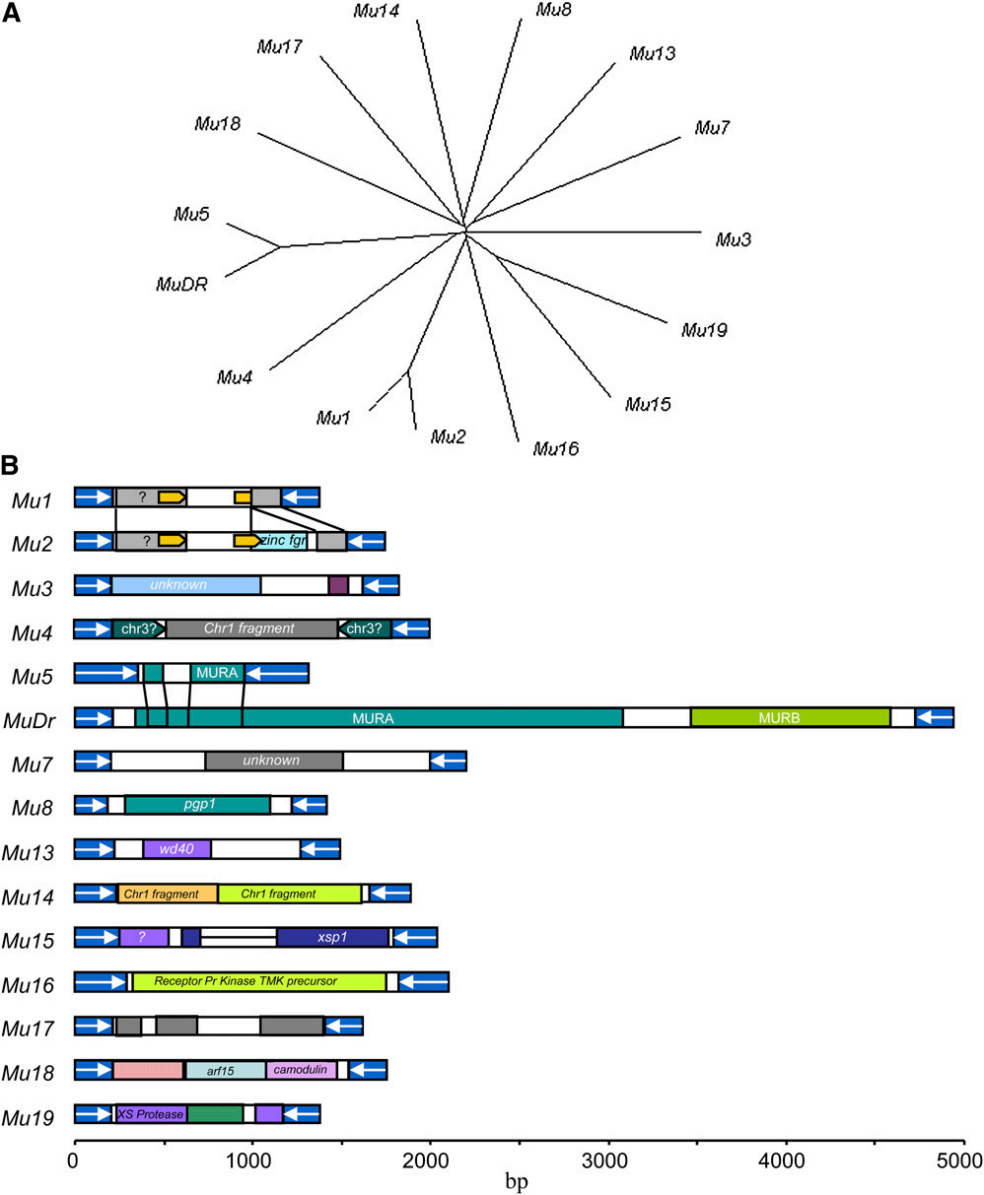
Sequence and structure of new *Mu* elements (*Mu13–Mu19*) and previous known *Mu* elements. (A) Phylogenetic tree derived by CLUSTALW by using the internal sequences of each *Mu* elements. (B) Schematic structure of each *Mu* element. Arrows indicate terminal inverted repeats. Internal captured gene fragments are labeled based on similarity to host genome. Refer to text for captured gene fragments.

A phylogenetic analysis performed by using the internal sequences indicated that these elements are not related, except for *Mu1* and *Mu2* (also known as *Mu1.7*), *Mu5*, and *MuDR* (Figure 5A). Sequence analysis strongly supports the notion that *Mu1* is a deletion derivative of *Mu2*, and that *Mu5* a deletion derivative of *MuDR*. *Mu2* contains a 140bp direct repeat in the internal sequence (Figure 5B, box arrows). Its 3′ region (from 893 to 1330bp) showed a 91% identity to maize and rice genomic sequences, suggesting possible gene fragment capture. The conceptual translation product of this region showed high similarity (80% identity) to Os05g0128200, which was annotated as zinc finger CCCH domain-containing protein 33 in rice. In sequence alignment with *Mu2*, *Mu1* lacks most of this region, but still retains a residual 41 bp of the likely captured fragment. Similarly, *Mu5* contains two segments of the *mudrA* gene that codes for MURA transposase (Figure 5B). *Mu15* showed slight similarity to *Mu19*, in which three short segments of the internal sequences shared some similarity, suggesting that the two elements are likely of the same origin. The divergent sequences indicated that deletion and insertion also occurred fairly long ago. It has been known that the internal sequences of *Mu* elements are likely captured gene fragments. The captured gene fragments were analyzed in the known *Mu* elements (Lisch 2002). Our analysis indicated that *Mu3* and *Mu4* can be classified as Pack-MULEs. A fragment from maize chromosome 6 accounted for most of the internal sequence of *Mu3*. Additionally, two fragments fused from maize chromosome 1 and 3 accounted for the internal sequence of *Mu4*.

We analyzed the internal sequences of new *Mu* elements identified in this work. As indicated in Figure 5B, the *Mu13* element contains two regions that showed high similarity to a WD40 protein. *Mu14* contains a fragment highly similar (89% identity) to a putative cucumisin-like serine protease on chromosome 1. *Mu15* contains a fragment that is similar to xylem serine proteinase 1 on chromosome 1 (LOC100281759). *Mu16* contains a fragment similar to a receptor protein kinase TMK precursor (95% identity, Accession no. BT054484) on chromosome 3. *Mu17* contains fragments from different chromosomes. *Mu18* contains a fragment of an auxin response factor 15 (ARF15) gene (Accession no. HM004530, 97% identity at nt level) and a calmodulin (LOC100286292, 98% identity at nt level). *Mu19* contains a fragment of a putative xylem serine proteinase 1 (Accession no. NM_001154679). All of these elements except *MuDR* are between 1.4 and 2.5 kb in length.

**Figure 6:**
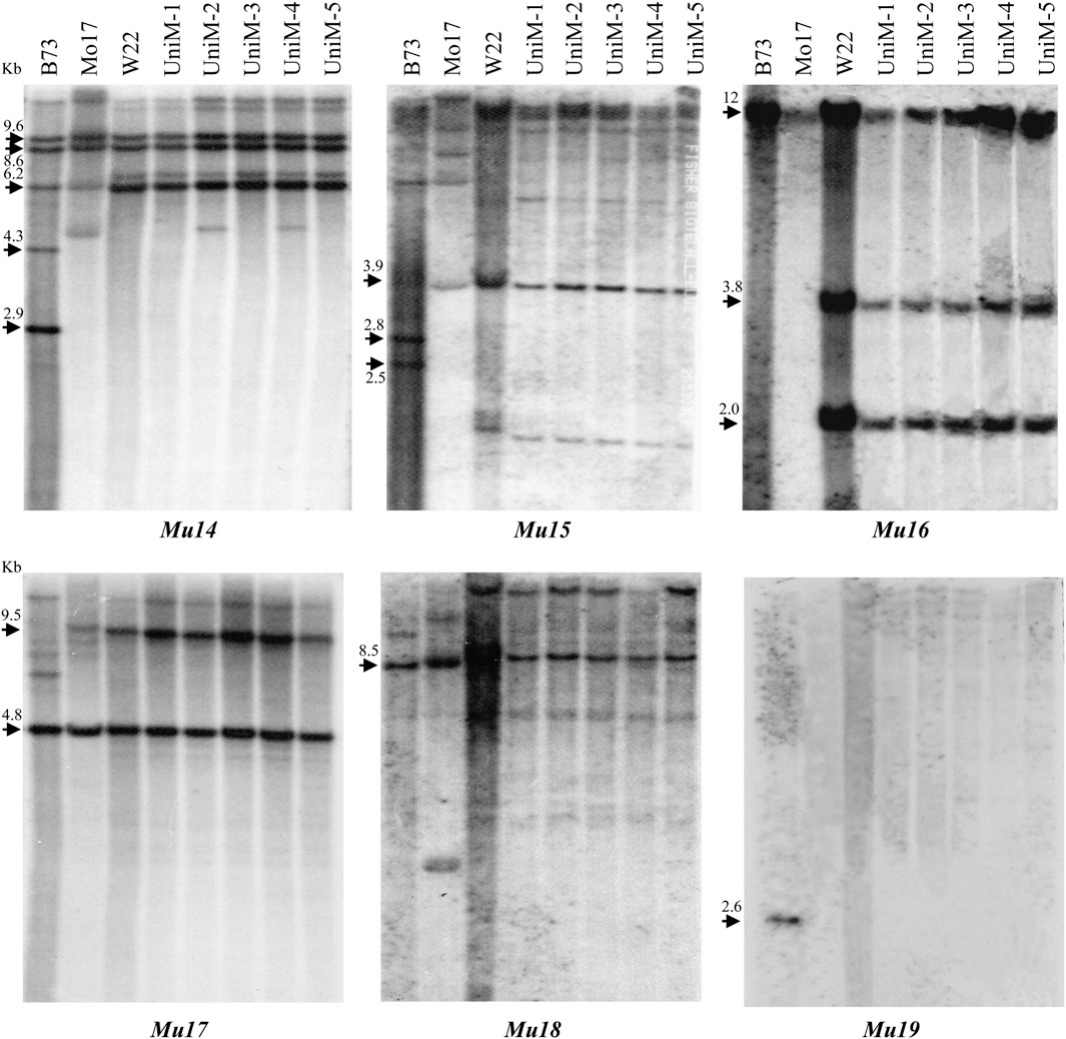
Presence of the new *Mu* elements in W22, Mo17, and the UniformMu line. Genomic DNAs were digested with *EcoRI* and probed with the internal sequence of each *Mu* element (as indicated underneath). UniM-1 to 5 were randomly selected mutant lines from the UniformMu population. Fragment sizes are indicated by arrows.

We cloned the internal sequences of these *Mu* elements and used them as probes to test their presence in inbred lines Mo17, W22, and six randomly selected UniformMu mutant lines. As shown in a Southern blot analysis (Figure 6), *Mu14* to *Mu18* elements were found in W22 and the UniformMu lines. The identical sizes of the fragments between W22 and the UniformMu lines strongly suggested that these elements were likely derived from W22. *Mu19* was not found in Mo17, W22, or the UniformMu lines, but was found in B73. The analysis revealed that these elements represent part of the non-colinear genome fraction of the three inbred lines. B73, Mo17, and W22 were all variable for these six elements in terms of copy numbers and RFLP size. *Mu19* was not present in either Mo17 or W22. It was also not detected in the six UniformMu lines. Some elements showed identical size among the three inbred lines, indicating likely early transposition events prior the separation of these inbreds. These *Mu* elements in the UniformMu population were derived from W22. Within the limited number of the UniformMu samples, new transposition events were not detected.

## Discussion

### *Mu13* is a new *Mu* transposable element

*Mutator* elements share a highly conserved ∼220 bp TIR sequence and create a 9bp TSD upon insertion (Walbot and Rudenko 2002). Different *Mu* elements are defined by the internal sequences between the TIRs. *Mu13* has a TIR of 223bp that is highly similar to the conserved TIR sequences of known *Mu* elements (Figure 1), and yet the internal sequence is completely different from known *Mu* elements. *Mu13* element was not found in the sequenced B73 genome, nor was it detected by Southern hybridization analysis (Figure 3). Of the two *Mu13* insertions identified in this study, each created a 9bp TSD. Hence, we concluded that *Mu13* is a new *Mu* element.

### *Mu13* contributes significantly to mutagenesis in the UniformMu population

Among the previously identified *Mu* elements, not all are equally active in transposition. *Mu* element transposition was driven by the autonomous element *MuDR* (Hershberger *et al.* 1991). *Mu4* and *Mu5* were found inactive (Talbert *et al.* 1989), which may likely be due to the absence of the *MuDR* element. However, in a large scale tagging of *gl8* locus, 58% insertions were caused by *Mu1*/*Mu2*, 25% by *MuDR*, 7% by *Mu11*, and the remaining elements (*Mu8*, *Mu12*, *Mu4* and *Mu10*) collectively merely contributed to 10% (Dietrich *et al.* 2002). Our analysis of the known *Mu* element transposition events in the UniformMu population showed somewhat different presentation. *Mu1* showed higher frequency of transposition, followed by *Mu3*, *Mu8*, and *MuDR* (B. C. Tan and D. R. McCarty, unpublished data).

We have provided evidence that *Mu13* is active by analyzing new transposition events in a random selection of the UniformMu mutant lines, as well as by identification of *Mu13* insertions in two functional genes. After the initial identification of *Mu13*, we recovered another *Mu13* insertion in one of two insertions analyzed. These results indicated that *Mu13* is highly active in transposition in the UniformMu population. Conceivably, if *Mu13* is present in other *Mu* active lines, it should contribute significantly to mutagenesis as well. All the *Mu* active lines were derived from a single line from which *Mu* transposable elements were discovered (Chandler and Hardeman 1992). The UniformMu population was derived by introgressing Robertson’s *Mu* active line into inbred W22. Robertson maintained the *Mu* activity by out-crossing with W23; thereby *Mu13* could have been derived from W23. However, our Southern blot analysis as well as PCR detection confirmed the presence of *Mu13* in W22. Comparison of the fragment sizes of *Mu13* inserted elements between W22 and the UniformMu lines indicated that they are identical. Unless W23 contained the similar *Mu13* insertions, the *Mu13* elements in the UniformMu population appeared to be derived from W22. Hence, it is possible that the introgression of *MuDR* elements from the *Mu* active line activated the *Mu13* element in the W22. It would be interesting to test the presence of the *Mu13* element in other mutagenesis populations, such as the Maize-targeted mutagenesis population (May *et al.* 2003) and the Pioneer Hi-Bred International’s Trait Utility System in Corn collection (Bensen *et al.* 1995).

### Unidentified *Mu* elements in the maize genome and their activity

The identification of active *Mu13* in the maize genome suggested that there are many more unknown *Mu* elements in the genome. The sequencing of the B73 genome recovered many of these elements (Schnable *et al.* 2009), but evidence suggested that there are more. We have analyzed six *Mu* elements identified in B73, and *Mu19* was not detected in the UniformMu population, which is largely W22 introgressed with the Robertson’s active *Mu* line. *Mu13* was not found in the B73 genome, and in the tagging of 80 alleles of *gl8*, *Mu13* was not detected in the population that derived from the Robertson’s Mu-active line. *Mu13* apparently was present in W22 and was activated during introgressing with Mu-active lines. The W22 line did not contain any active *MuDR* element (B. C. Tan and D. R. McCarty, unpublished data), hence *Mu13* was inactive. Because different maize inbred lines harbor different spectrums of *Mu* elements, more unknown *Mu* elements are expected. It is highly likely that most of the maize inbred lines did not contain any active *MuDR* elements, hence all the *MuDR* driven *Mu* elements are dormant. Upon introducing the *MuDR* element, *Mu* element activity may be restored. If this is the case, Southern blot based cosegregation analysis using known *Mu* internal sequences as probes may encounter some problems. But this will not affect analysis based on the TIR sequences such as AIMS (Frey *et al.* 1998), Mu-TAIL PCR (Settles *et al.* 2004), AIMS and Mu-TAIL-PCR combined (Yi *et al.* 2009), and the use of PCR-coupled with pyrosequencing (Williams-Carrier *et al.* 2010). In addition, if the creation of new *Mu* elements is associated with the *MuDR* activity, it will be expected that there will be many new *Mu* elements in Robertson’s Mu-active line. It will be interesting to know the *Mu* landscape in the Robertson *Mu* active line.

## References

[bib1] Bennetzen J. L., 1984 Transposable element *Mu1* is found in multiple copies only in Robertson’s Mutator maize lines. J. Mol. Appl. Genet. 2: 519–524 6099399

[bib2] Bennetzen J. L. Swanson J. Taylor W. C. Freeling M., 1984 DNA insertion in the first intron of maize *Adh1* affects message levels: cloning of progenitor and mutant *Adh1* alleles. Proc. Natl. Acad. Sci. USA 81: 4125–4128 6330742 10.1073/pnas.81.13.4125 PMC345381

[bib3] Bensen R. J. Johal G. S. Crane V. C. Tossberg J. T. Schnable P. S., 1995 Cloning and characterization of the maize *An1* gene. Plant Cell 7: 75–84 7696880 10.1105/tpc.7.1.75 PMC160766

[bib4] Chalvet F. Grimaldi C. Kaper F. Langin T. Daboussi M. J., 2003 Hop, an active Mutator-like element in the genome of the fungus *Fusarium oxysporum*. Mol. Biol. Evol. 20: 1362–1375 12777515 10.1093/molbev/msg155

[bib5] Chandler V. L. Hardeman K. J., 1992 The Mu elements of Zea mays. Adv. Genet. 30: 77–122 1333722 10.1016/s0065-2660(08)60319-3

[bib6] Chen C. H. Oishi K. K. Kloeckener-Gruissem B. Freeling M., 1987 Organ-specific expression of maize *Adh1* is altered after a *Mu* transposon insertion. Genetics 116: 469–477 3038674 10.1093/genetics/116.3.469 PMC1203158

[bib7] Dietrich C. R. Cui F. Packila M. L. Li J. Ashlock D. A., 2002 Maize *Mu* transposons are targeted to the 5′ untranslated region of the *gl8* gene and sequences flanking *Mu* target-site duplications exhibit nonrandom nucleotide composition throughout the genome. Genetics 160: 697–716 11861572 10.1093/genetics/160.2.697 PMC1461997

[bib8] Eisen J. A. Benito M. I. Walbot V., 1994 Sequence similarity of putative transposases links the maize Mutator autonomous element and a group of bacterial insertion sequences. Nucleic Acids Res. 22: 2634–2636 8041625 10.1093/nar/22.13.2634 PMC308220

[bib9] Fleenor D. Spell M. Robertson D. Wessler S., 1990 Nucleotide sequence of the maize Mutator element, *Mu8*. Nucleic Acids Res. 18: 6725 2251152 10.1093/nar/18.22.6725 PMC332669

[bib10] Frey M. Stettner C. Gierl A., 1998 A general method for gene isolation in tagging approaches: amplification of insertion mutagenised sites (AIMS). Plant J. 13: 717–721

[bib11] Hershberger R. J. Warren C. A. Walbot V., 1991 Mutator activity in maize correlates with the presence and expression of the Mu transposable element *Mu9*. Proc. Natl. Acad. Sci. USA 88: 10198–10202 1719548 10.1073/pnas.88.22.10198 PMC52895

[bib12] Hershberger R. J. Benito M. I. Hardeman K. J. Warren C. Chandler V. L., 1995 Characterization of the major transcripts encoded by the regulatory *MuDR* transposable element of maize. Genetics 140: 1087–1098 7672579 10.1093/genetics/140.3.1087 PMC1206663

[bib13] Hua-Van A. Capy P., 2008 Analysis of the DDE motif in the Mutator superfamily. J. Mol. Evol. 67: 670–681 19018586 10.1007/s00239-008-9178-1

[bib14] Jiang N. Bao Z. Zhang X. Eddy S. R. Wessler S. R., 2004 Pack-MULE transposable elements mediate gene evolution in plants. Nature 431: 569–573 15457261 10.1038/nature02953

[bib15] Juretic N. Hoen D. R. Huynh M. L. Harrison P. M. Bureau T. E., 2005 The evolutionary fate of MULE-mediated duplications of host gene fragments in rice. Genome Res. 15: 1292–1297 16140995 10.1101/gr.4064205 PMC1199544

[bib16] Lisch D. R. Freeling M. Langham R. J. Choy M. Y., 2001 Mutator transposase is widespread in the grasses. Plant Physiol. 125: 1293–1303 11244110 10.1104/pp.125.3.1293 PMC65609

[bib17] Lisch D., 2002 Mutator transposons. Trends Plant Sci. 7: 498–504 12417150 10.1016/s1360-1385(02)02347-6

[bib18] Lisch D., 2005 Pack-MULEs: theft on a massive scale. Bioessays 27: 353–355 15770680 10.1002/bies.20219

[bib19] Matsuoka Y. Vigouroux Y. Goodman M. M. Sanchez G. J. Buckler E., 2002 A single domestication for maize shown by multilocus microsatellite genotyping. Proc. Natl. Acad. Sci. USA 99: 6080–6084 11983901 10.1073/pnas.052125199 PMC122905

[bib20] May B. P. Liu H. Vollbrecht E. Senior L. Rabinowicz P. D., 2003 Maize-targeted mutagenesis: A knockout resource for maize. Proc. Natl. Acad. Sci. USA 100: 11541–11546 12954979 10.1073/pnas.1831119100 PMC208794

[bib21] McCarty D. R. Settles A. M. Suzuki M. Tan B. C. Latshaw S., 2005 Steady-state transposon mutagenesis in inbred maize. Plant J. 44: 52–61 16167895 10.1111/j.1365-313X.2005.02509.x

[bib22] Pritham E. J. Feschotte C. Wessler S. R., 2005 Unexpected diversity and differential success of DNA transposons in four species of entamoeba protozoans. Mol. Biol. Evol. 22: 1751–1763 15901838 10.1093/molbev/msi169

[bib23] Raizada M. N., 2003 RescueMu protocols for maize functional genomics. Methods Mol. Biol. 236: 37–58 14501057 10.1385/1-59259-413-1:37

[bib24] Robertson D. S., 1978 Characterization of a mutator system in maize. Mutat. Res. 51: 21–28

[bib25] Robertson D. S., 1981 Mutator activity in maize: timing of its activation in ontogeny. Science 213: 1515–1517 17780881 10.1126/science.213.4515.1515

[bib26] Saccaro N. L. Jr. Van Sluys M. A. de Mello V. A. Rossi M., 2007 MudrA-like sequences from rice and sugarcane cluster as two bona fide transposon clades and two domesticated transposases. Gene 392: 117–125 17289300 10.1016/j.gene.2006.11.017

[bib27] Schnable P. S. Ware D. Fulton R. S. Stein J. C. Wei F., 2009 The B73 maize genome: complexity, diversity, and dynamics. Science 326: 1112–1115 19965430 10.1126/science.1178534

[bib28] Settles A. M. Latshaw S. McCarty D. R., 2004 Molecular analysis of high-copy insertion sites in maize. Nucleic Acids Res. 32: e54 15060129 10.1093/nar/gnh052 PMC390377

[bib29] Talbert L. E. Patterson G. I. Chandler V. L., 1989 *Mu* transposable elements are structurally diverse and distributed throughout the genus *Zea*. J. Mol. Evol. 29: 28–39 2549259 10.1007/BF02106179

[bib30] Taylor L. P. Walbot V., 1987 Isolation and characterization of a 1.7-kb transposable element from a mutator line of maize. Genetics 117: 297–307 10.1093/genetics/117.2.297 PMC1203205 2444493

[bib31] Walbot V. Rudenko G. N., 2002 *MuDR/Mu* transposable elements of maize, pp. 533–564 Moble DNA II, edited by Craig N. L. Craigie R. Gellert M. Lambowitz A. M. ASM Press, Washington, DC

[bib32] Williams-Carrier R. Stiffler N. Belcher S. Kroeger T. Stern D. B., 2010 Use of Illumina sequencing to identify transposon insertions underlying mutant phenotypes in high-copy Mutator lines of maize. Plant J. 63: 167–177 20409008 10.1111/j.1365-313X.2010.04231.x

[bib33] Xu Z. Yan X. Maurais S. Fu H. O’Brien D. G., 2004 Jittery, a Mutator distant relative with a paradoxical mobile behavior: excision without reinsertion. Plant Cell 16: 1105–1114 15075398 10.1105/tpc.019802 PMC423203

[bib34] Yi G. Luth D. Goodman T. D. Lawrence C. J. Becraft P. W., 2009 High-throughput linkage analysis of *Mutator* insertion sites in maize. Plant J. 58: 883–892 19207214 10.1111/j.1365-313X.2009.03821.x

